# Gastrointestinal Spatiotemporal mRNA Expression of *Ghrelin* vs *Growth Hormone Receptor* and New Growth Yield Machine Learning Model Based on Perturbation Theory

**DOI:** 10.1038/srep30174

**Published:** 2016-07-27

**Authors:** Tao Ran, Yong Liu, Hengzhi Li, Shaoxun Tang, Zhixiong He, Cristian R. Munteanu, Humberto González-Díaz, Zhiliang Tan, Chuanshe Zhou

**Affiliations:** 1 Key Laboratory for Agro-Ecological Processes in Subtropical Region, Hunan Research Center of Livestock & Poultry Sciences, South-Central Experimental Station of Animal Nutrition and Feed Science in Ministry of Agriculture, Institute of Subtropical Agriculture, Chinese Academy of Sciences (CAS), Changsha, Hunan, 410125, P. R. China; 2 Graduate University of Chinese Academy of Sciences, Beijing 100049, P. R. China; 3 RNASA, Computer Sciences Faculty, University of A Coruna, Campus de Elviña s/n, 15071, A Coruña, Spain; 4 Department of Organic Chemistry II, University of the Basque Country UPV/EHU, 48940, Leioa, Spain; 5 IKERBASQUE, Basque Foundation for Science, 48011, Bilbao, Spain

## Abstract

The management of ruminant growth yield has economic importance. The current work presents a study of the spatiotemporal dynamic expression of *Ghrelin* and *GHR* at mRNA levels throughout the gastrointestinal tract (GIT) of kid goats under housing and grazing systems. The experiments show that the feeding system and age affected the expression of either Ghrelin or GHR with different mechanisms. Furthermore, the experimental data are used to build new Machine Learning models based on the Perturbation Theory, which can predict the effects of perturbations of *Ghrelin* and *GHR* mRNA expression on the growth yield. The models consider eight longitudinal GIT segments (rumen, abomasum, duodenum, jejunum, ileum, cecum, colon and rectum), seven time points (0, 7, 14, 28, 42, 56 and 70 d) and two feeding systems (Supplemental and Grazing feeding) as perturbations from the expected values of the growth yield. The best regression model was obtained using Random Forest, with the coefficient of determination R^2^ of 0.781 for the test subset. The current results indicate that the non-linear regression model can accurately predict the growth yield and the key nodes during gastrointestinal development, which is helpful to optimize the feeding management strategies in ruminant production system.

Ghrelin has been drawing researchers’ attention due to its multiple functions since it was discovered by Kojima *et al*. in the rat stomach extracts in 1999^1^, and it has been widely studied in human and rodents since then^2^. Ghrelin, a 28-amino acid peptide, is post translationally octanoylated by the ghrelin-O-acyl transferase to bind and activate its cognate receptor, the growth hormone secretagogue receptor-1a (*GHSR-1a*)^3^. Ghrelin has been initially validated as an endogenous ligand for the *GHSR-1a* (also known as Ghrelin receptor), and it plays a role in regulating the growth hormone (GH) release^1^. GH regulates numerous cellular functions by direct binding to its receptors, growth hormone receptors (GHR), in various tissues^4^. In the case of the gastrointestinal tracts (GIT), it has been proven that GH has proliferative effects on the intestinal epithelium, and it influences enteroendocrine cell secretion, calcium absorption, and intestinal amino acid and ion transport^5^. However, a series of studies have provided compelling evidence to shift the focus to ghrelin as a regulator energy homeostasis^6^,^7^,^8^, accomplished by affecting appetite^9^, food intake^10^, and body weight^11^,^12^. Over the past decades, Ghrelin has been viewed as a central modulator of energy homeostasis, due to its ability of increasing GH secretion and stimulating food intake^12^. Nowadays, Ghrelin’s role in energy homeostasis is generally perceived as its most important function^11^,^12^.

Ghrelin has also been proven to be secreted in the stomach of domestic ruminant animals (cow and goat) shortly after the discovery of Ghrelin^13^. Until recently, Ghrelin has been purified and characterized from the stomach of Caprinae, a 27 amino acid peptide that lacks Gln^14^. Some studies have focused on possible roles Ghrelin played in ruminants^13^,^15^,^16^,^17^,^18^,^19^,^20^. The GHR has also been widely studied in ruminants, and it has been suggested as a preferential target for genetic breeding because it is significantly associated with growth traits, such as milk yield, meat production and growth^21^,^22^,^23^,^24^. As stated in a previous study^25^, the Ghrelin pathway (which includes Ghrelin, GHSR-1a, and ghrelin-O-acyl transferase) is a potential target for the improvement of ruminants’ production. This could be achieved through the modification of feeding behavior, body composition, immune and reproductive functions.

The growth of young goats is generally separated into three phases according to the rumen development process: pre-rumination (0–3 weeks), transition (3–8 weeks) and rumination phases (from 8 weeks ahead)^26^. Nowadays, two predominant feeding patterns (supplemental feeding and grazing) are widespread throughout the world in ruminant production: in intensive farming, supplemental feeding is a preferred method of providing nutrients with emphasis on offering young ruminants a solid starter concentrate at a relative early age; however, ruminant production is based primarily on grazed pastures with limited supplemental feeding in many underdeveloped areas of the world^27^. Accordingly, young ruminants face a transition in feed supply from milk, during the pre-ruminant suckling phase, to supplemental feeding of starter concentrate in intensive farming or to poorly digestible grazing pastures in traditional farming during the transitional phase. Unfortunately, there is a lack of knowledge on *Ghrelin* and *GHR* mRNA expression under supplemental feeding and grazing conditions. Since the Ghrelin axis plays important roles in energy homeostasis, it is of interest to study the dynamic developmental changes of *Ghrelin* and *GHR* mRNA expression during different stages of development under Supplemental feeding vs Grazing (S vs G) feeding systems.

Perturbation Theory (PT) is a mathematical method used to search for an approximate solution of a problem, by dividing a complex problem into smaller “solvable” and “perturbation” parts. In other words, PT deals with a specific problem by adding corrections or “perturbations” according to the variations of different experimental conditions (c_j_). In general, this theory is described as a function f(δ_i_) for outputs of the predictive model that constructed using variables/features/properties (δ_i_) in a system under a set of experimental conditions (c_j_)^28^. In our previous studies, Moving Average (MA) has been used to measure the deviations of the different input variables in PT models for molecular bio-systems^29^,^30^,^31^, as well as binary micelle nanoparticles^32^. Thus, MA makes a time series stationary using these deviations as differences. The combination of PT and MA/Box-Jenkins Operators was found to be useful in making predictive models for growth yield, Y(ζ_k_). This model is based on the expression of *Ghrelin* and *GHR* under S vs G feeding systems. There are three objectives of the current study: (1) to investigate the tissue distribution and sequential dynamic developmental changes of *Ghrelin* and *GHR* mRNA expression during different stages of development; (2) to determine the effect of S vs G feeding systems on the mRNA expression of *Ghrelin* and *GHR*; (3) to build a new machine learning model of Y(ζ_k_) based on the expression of *Ghrelin* and *GHR* under S vs G feeding systems.

## Results and Discussion

### Spatiotemporal mRNA expression of Ghrelin and GHR

**Figure 1:**
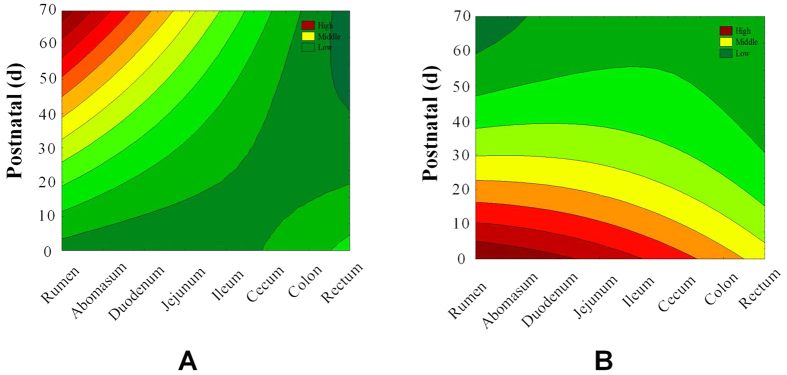
The predicted spatiotemporal mRNA expression of *Ghrelin* (**A**) and *GHR* (**B**) throughout the gastrointestinal tract (GIT) of kid goats.

During all the three stages of development, *Ghrelin* was expressed throughout the GIT of kid goats, with a greater expression of *Ghrelin* (*P* < 0.01) observed in the abomasum than those in the other remaining segments (Table 1). This was in accordance with a previous study in sheep^18^. The predominant expression of *Ghrelin* mRNA in the abomasum was consistent with the findings observed in humans and rats. As the abomasum of ruminants is functionally like the stomach of non-ruminants, the predominant expression of *Ghrelin* mRNA in the abomasum of ruminants was also consistent with the greater expression of *Ghrelin* in the stomach of non-ruminants (humans and rodents)^1^,^33^,^34^. This implied that similar organs evolved according to similar working mechanisms. The *Ghrelin* was moderately expressed in the duodenum and jejunum. This was consistent with the results of *Ghrelin* expression in the small intestine of humans and rats^34^,^35^. Meanwhile, the expression of *Ghrelin* in the abomasum was increased (*P* < 0.001) with age (from pre-rumination phase to rumination phase); however, the mRNA expression of *Ghrelin* in the other segments was almost unchanged (*P* > 0.05) with age (Table 1). A more intuitional predicted presentation of spatiotemporal mRNA expression of *Ghrelin* throughout the GIT of kid goats is shown in Fig. 1A. A similar sequential, dynamic, and developmental change of *Ghrelin* was also observed in sheep by Huang *et al*.^18^, in whose study the *Ghrelin* mRNA levels were gradually increased in the abomasum in accordance with growth curve during early developmental periods.

Like *Ghrelin*, *GHR* was expressed throughout the GIT of kid goats during three developmental stages (Table 2), and a more intuitional predicted presentation of spatiotemporal mRNA expression of *GHR* is shown in Fig. 1B. These results are in agreement with previous studies carried out in the GIT of humans and rats^36^,^37^. The widespread expression of *GHR* in the GIT suggested the regulatory roles of GH on digestive and immune functions, including metabolism, growth, or differentiation^5^,^38^. During pre-rumination phase, the expression of *GHR* in the abomasum, duodenum and jejunum was greater (*P* < 0.01) than those in the other GIT segments; similarly, *GHR* tissue distribution pattern was observed during transition and rumination phases, with relatively greater expression in the abomasum, followed by the duodenum, colon and rectum. Furthermore, the expression of *GHR* in major segments of GIT (except for the colon and rectum) was reduced (*P* < 0.05) with age (Table 2). In porcine gastric tissue, the gastric *GHR* mRNA expression was found to be significantly correlated with the relative gastric weight (r = 0.541)^39^.

### Effects of feeding systems on mRNA expression of Ghrelin and GHR

During time interval d 28–70, the mRNA expression of *Ghrelin* in the abomasum, duodenum and jejunum was affected (*P* < 0.01) by both feeding system and age, with relatively greater expression in the G group than those in the S group (Table 1). The expression of *GHR* at mRNA level was affected by the feeding system (*P* ≤ 0.001) in all segments of the GIT and by age (*P* < 0.05) in major segments of GIT (except for the jejunum and rectum), with greater expression in the S group than in the G group in all GIT segments (Table 2). This suggested that the expression of *Ghrelin* and *GHR* was reversely affected by feeding type and age. Sugino *et al*.^15^ reported that the expression of *Ghrelin* can be modified by the feeding regimen in sheep; the Ghrelin secretion levels before prandium are higher in animals fed twice daily than those in animals fed four times daily^40^. Since Ghrelin usually acts as a starvation signal, it was reasonable that reduced *Ghrelin* mRNA expression was observed in the S group supplemented with concentrates in the current study. It has long been accepted that the *GHR* abundance is submitted to a developmental and nutritional regulation in a tissue-specific manner^4^. However, few works studied the *GHR* expression in the GIT under different nutritional status. The present results implied that supplemental feeding could increase the *GHR* mRNA expression in the GIT. However, there were no feeding system × age interactions (*P* > 0.05) in the GIT segments on either *Ghrelin* or *GHR* expression, except for *Ghrelin* expression in the duodenum (*P* < 0.01) and *GHR* expression in the rumen (*P* < 0.001) and jejunum (*P* < 0.05). This further implied that feeding system and age affected the expression of either *Ghrelin* or *GHR* with different mechanisms. Furthermore, from time points d 0 to 70, the expression of *Ghrelin* in the abomasum was quadratically increased. The same increase pattern in *Ghrelin* expression was also observed in the duodenum (both S and G groups) and jejunum. The expression of *GHR* in the rumen and duodenum was affected by age, with a linear decrease for both S and G groups (*P* < 0.01), as well as a quadratic decrease for the G group (*P* < 0.01). In the abomasum, jejunum, ileum and cecum, the expression of *GHR* at mRNA level was affected by age linearly (*P* < 0.01); the quadratic decrease was observed in the jejunum, ileum, cecum and colon (*P* < 0.05).

### Model construction

#### Dataset

The present study first uses reported data of the *Ghrelin* and *GHR* gene mRNA expression throughout the GIT of kid goats, and our previous work data^27^ of Live weight vs Carcass weight (L_w_ vs C_w_) that were employed as a dataset for Y(ζ_k_) model construction. Different experimental conditions such as the longitudinal GIT segments (s), the postnatal time (t), and the feeding method (m) were defined as deviations or perturbations of the Y(ζ_k_) model (where ζ = s, t, and m). Each perturbation had several levels (k), expressed as k′, k″ and k″′, respectively. In detail, s_k′_ represents 8 different segments of the GIT (k′ = 1, 2, 3, 4, 5, 6, 7 and 8; 1 = rumen, 2 = abomasum, 3 = duodenum, 4 = jejunum, 5 = ileum, 6 = cecum, 7 = colon and 8 = rectum). t_k″_ represents 7 different sampling time points ranging from d 0 to 70 postnatal (k″ = 1, 2, 3, 4, 5, 6 and 7, and represents d 0, 7, 14, 28, 42, 56 and 70 postnatal). m_k″′_ means 2 different feeding systems (k″′ = 1 and 2, and refers to Supplemental feeding and Grazing). A total of samples N_s_ = 352 collected from 44 (N_a_ = 44, N_a_ = number of experimental animals) kid goats were studied for the mRNA expression of *Ghrelin* and *GHR*. The detailed full dataset was provided in online supplementary material SM01^41^. In order to carry out a perturbation theory analysis, a pair-wise analysis of query samples vs reference samples was done by using a previous dataset of N_s_ to construct a two-block dataset (see SM01^41^), with the number of perturbation cases N_c_ = 123 872 pairs of query and reference samples selected randomly from the 352 samples. The details about PT models could be found in our previous works^28^,^42^.

#### Regression models

**Figure 2:**
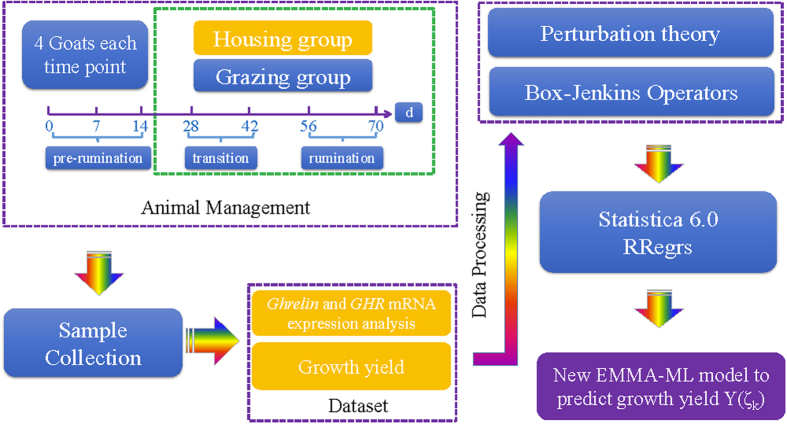
Flow chart of experimental and theoretical sections for Y(ζk) predictive models.

The schematic diagram of the present work aimed at developing a new Expected Measurement Moving Average–Machine Learning (EMMA-ML) model to predict growth yield Y(ζ_k_) is presented in Fig. 2. Box-Jenkins Operators and PT were used to handle the deviations or perturbations in the current study. In addition, Moving Average (MA) was also used to account for the “small” deviations of different growth stages, the spatiotemporal factors on the *Ghrelin* and *GHR* mRNA expression.

Several types of predictive model were presented in the study for growth ratio–yield Y(ζ_k_) based on the perturbation or variations of different experimental conditions (ζ_k_ = s_k′_, t_k″_, m_k″′_). In the first step, Y(ζ_k_)_exp_ was defined as the expected Y(ζ_k_) values under a set of given experimental conditions (ζ_k_ = s_k′_, t_k″_, m_k″′_). Then, MA was used to define the “small” deviations under the different conditions (ζ_k_). Finally, a general formula of EMMA models for predicted growth yield Y(ζ_k_)_pred_. Eq. 1 showed the linear model constructed by setting the mRNA expression of *Ghrelin* and *GHR* as variables input.


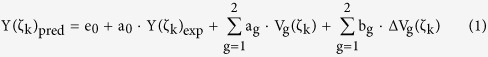


Y(ζ_k_)_pred_ represents the predicted value of Y(ζ_k_). The coefficients for each input variables in this general equation are e_0_, a_0_, a_g_ and b_g_. The subscript “g” refers to two input variables: the mRNA expression of *Ghrelin* (g = 1) and *GHR* (g = 2). The expected value of Y(ζ_k_), Y(ζ_k_)_exp_, is the first class of input variables. V_g_(ζ_k_) represents the mRNA expression of *Ghrelin* or *GHR*, the second class of input variables. ΔV_g_(ζ_k_) are the perturbation values and the third class of input variables. In Eq. 2, the general equation was expanded according to a set of experimental conditions (ζ_k_).


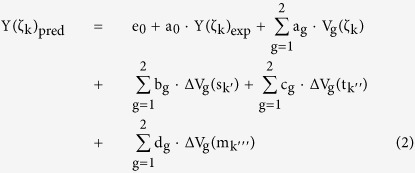


e_0_, a_0_, a_g_, b_g_, c_g_ and d_g_ are the coefficients for the corresponding input variables and Y(ζ_k_)_exp_ represents the expected values of Y(ζ_k_) in a set of reference experimental conditions. V_g_(ζ_k_) represents the input variables in a query (set of conditions) (ζ_k_ = s_k′_, m_k″_, or t_k″′_, respectively). Another class of input variables, ΔV_g_(s_k′_), ΔV_g_(t_k″_) and ΔV_g_(m_k″′_), refers the perturbation values in a set of experimental conditions s_k″_, t_k″_ and m_k″′_ for each V_g_(ζ_k_). <V_g_(ζ_k_)> is the MA/Box-Jenkins Operator for variable V_g_(ζ_k_) (Eq. 3), which can be calculated with all the perturbation cases under the same experimental conditions^28^,^43^.


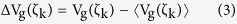


Thus, Y(ζ_k_)_exp_, V_g_(ζ_k_) and ΔV_g_(ζ_k_) were employed as input variables to develop a new Machine Learning predictive model using Statistica 6.0^44^ and RRegrs package^45^,^46^.

#### Mapping Ghrelin/GHR vs Yield

The first tested method was the General Multilinear Regression (GRM) from STATISTICA. The model predicted the effects of spatiotemporal perturbations of *Ghrelin* and *GHR* mRNA expression on Y(ζ_k_) and it is presented in Eq. 4. For the sake of simplicity, the output/features have the compacted notations in the model analysis: Y_pred_ = Y(ζ_k_), Y_exp_ = Y(ζ_k_)_exp_, V_1_ = V_1_(ζ_k_), dV_2_sk = ΔV_2_(s_k′_), dV_2_tk = ΔV_2_(t_k″_), and dV_2_mk = ΔV_g_(m_k″′_). This model was trained/validated with a total of N_c_ = 123 872 cases of perturbations (supplementary material SM02^47^). The full dataset of input variables and output values used to develop the new model were published in online supplementary material SM03^48^.


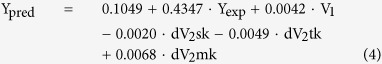


The statistics of this model are N = 123872, R = 0.733, R^2^ = 0.537, SS Residual = 920.012, df = 5, F = 21520.084 with *P* < 0.0001. The *P*-values for only the first two features (Y_exp_ and V_1_) are less than 0.05. The rest of the features are less important for the model (see details in SM03^48^). R represents the regression coefficient; R^2^ is the coefficient of determination; SS Residual is the residual sum of squares. The model obtained significant values (*P *<* 0.05*) with a low R value that could be considered as acceptable^49^.

There are five input variables of GRM model: the expected value of growth yield Y_exp_ for a set of given conditions, the levels of mRNA expression of *Ghrelin* gene V_1_ and three variables accounted for perturbations of *GHR* mRNA (dV_2_sk), deviations due to the changes of animal management (dV_2_mk), and postnatal time dV_2_tk. This model indicated that all the experimental conditions played different roles in Ypred. Additionally, all the expected values of mRNA expression of *GHR* gene <V_2_(ζ_k_)> (different segments, growth stages and feeding systems) influenced the growth yield. More specifically, the growth yield Ypred was increased with the increasing mRNA expression of expected values <V_2_(s_k′_)> and <V_2_(t_k″_)> for different GIT segments and age, but the growth yield was decreased with the expected values of <V_2_(m_k″′_)> for different types of feeding management. Expected values <V_2_(ζ_k_)> of *GHR* mRNA expression corresponding to the growth yields are shown in Table 3. In addition, the mRNA expression of *GHR* in the abomasum and duodenum could positively increase the growth yield. This was in accordance with previous reports claiming that GH has proliferative effects on the intestinal epithelium, and influences enteroendocrine cell secretion, calcium absorption, and intestinal amino acid and ion transport^5^. The *P*-values of the features show that only Y_exp_ and V_1_ are important for the linear model. Thus, the linear model shows that mRNA expression of *Ghrelin* is more important for the growth yield compared to the perturbations of *GHR* mRNA expression. Thus, it can be stated that the perturbations of *GHR* mRNA expression need non-linear modelling for the growth yield production.

In addition to the GLM method from STATISTICA, two types of neural networks were tested with the normalized dataset (see Table 4): Linear Neural Network (LNN, no hidden layers) and Multilayer Perceptron (MLP, with at least one hidden layer). Both models presented R^2^_test_ values between 0.529 and 0.539. The models have the same problem as the GRM one; the perturbations improve a little the model performance: LNN − > MLP with 1 hidden layer − > MLP with 2 hidden layers (with the same number of inputs). The best MLP model was MLP 5:5-15-12-1:1: 5 inputs, 15 neurons in the hidden layer 1, 12 neurons in the hidden layer 2, R^2^_test_ = 0.539. As expected, LNN 5:5–1:1 is the linear combination of the input features and it showed similar results to GRM with R^2^ = 0.534 (see details in SM03^48^).

In order to test different complex regression methods, seven regression RRegrs methods^45^,^46^ were used to build prediction models: Multiple Linear regression (LM), Generalized Linear Model with Stepwise Feature Selection (GLM)^50^, Partial Least Squares Regression (PLS)^51^, Lasso regression (Lasso)^52^, Elastic Net regression (ENET)^53^, single hidden layer Neural Networks regression (NN)^54^, and Random Forest regression (RF)^55^. Table 5 shows RMSE and R^2^ values for the training and test subsets. LM, GLM PLS, Lasso and ENET provided models close to the GLM from STATISTICA: R^2^_test_ = 0.533–0.534 and RMSE_test_ = 0.0992–0.0994. Lasso is not able to provide a model that includes the system perturbations and it used only one feature, Y_exp_. ENET was based on only two features (Y_exp_ and V_1_) with the same R^2^_test_ as LM/GLM.

**Figure 3:**
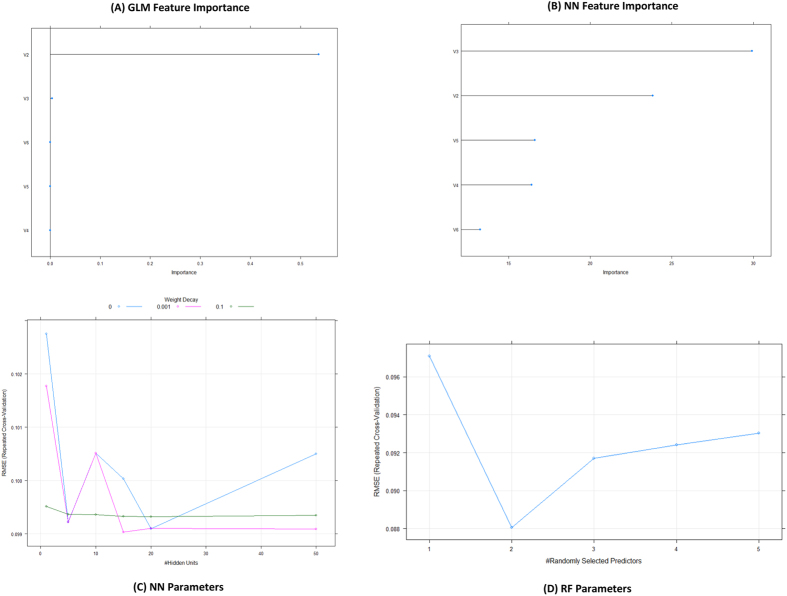
Statistical analysis of GLM, NN and RF models for the normalized dataset: (**A**) GLM feature importance, (**B**) NN feature importance, (**C**) NN parameter analysis, and (**D**) RF parameter analysis.

The feature importance analysis for GLM (Fig. 3A) pointed out the natural importance of Y_exp_ and the preference of using Y_exp_ and V_1_, obtaining similar results to those obtained with MLP in STATISTICA. Similar Y_exp_ feature importance is presented by Lasso and ENET. NN with one hidden layer provided a very small improvement, but with the same power as MLP 5:5-15-12-1:1 with 2 hidden layers from STATISTICA. Figure 3B shows the NN feature importance. Several parameters were tested to find the optimal topology of NN (see Fig. 3C): number of neurons = [1,5,10,15,20,50], weight decay = [0.0, 0.1, 0.001]. The optimal NN model 5-15-1 with the minimum RMSE_test_ value has all 5 input features, 15 neurons hidden layer neurons, a weight decay of 0.001, a higher R^2^_test_ value of 0.540 and lower RMSE_test_ of 0.0986 (see SM03^48^).

The best regression performance was obtained with the RF regression method: R^2^_test_ of 0.629 and RMSE_test_ of 0.0886 using five trees and all features. The variation of the RMSE_test_ with the number of features selected as inputs for the trees (RF parameter mtry) is presented in Fig. 3D. Thus, two features were used as input for each tree.

These statistics show the difficulty of the linear models to establish a relationship between the output and the features. The strong preference for Y_exp_ is natural because it was calculated based on the observable output values. Thus, the linear models have difficulty to include perturbation of V_2_ (*GHR* mRNA expression) in the model and only RF method was able to improve the regression model performance with R^2^_test_ > 0.60 (still low performance).

The results obtained with STATISTICA and RRegrs for the linear and non-linear regression models demonstrated the relatively moderate prediction power of the models using the original dataset, with a maximum of 0.629 for R^2^_test_ (RF): the model explains only 62.9% of the response data variability around its mean. For this reason, a pre-processing of the original datasets was used to improve the dataset quality by removing the outliers. Calculating the Pearson residuals for the fitted values, a filter was used for the cases with residuals which are twice (0.4) as high as the maximum residual of the majority of data (0.2). Therefore, only 2.3% of the cases were filtered, resulting in a new dataset of 121,056 cases. The raw and normalized corrected datasets were the inputs for the same linear and non-linear regression methods from STATISTICA and RRegrs tools.

In the first step, STATISTICA neural networks were tested for the normalized filtered dataset (see Table 6 and SM03^48^ for details). LNN and MLP (one and two hidden layers) predictors demonstrated improved regression performance. LNN 5:5-1:1 is the equivalent of a linear model using all five features and it provided improved R^2^_test_ of 0.774, only slightly superior to the non-filtered dataset (R^2^_test_ 0.534). MLP 2:2-11-1:1 with two features (Y_exp_, V_1_), 2 neurons in the first hidden layer and 11 neurons in the second hidden layer presents R^2^_test_ of 0.704. This performance is superior to the non-filtered dataset results such as MLP 5:5-15-12-1:1 with R^2^_test_ = 0.539, and RF with R^2^_test_ = 0.629.

**Figure 4:**
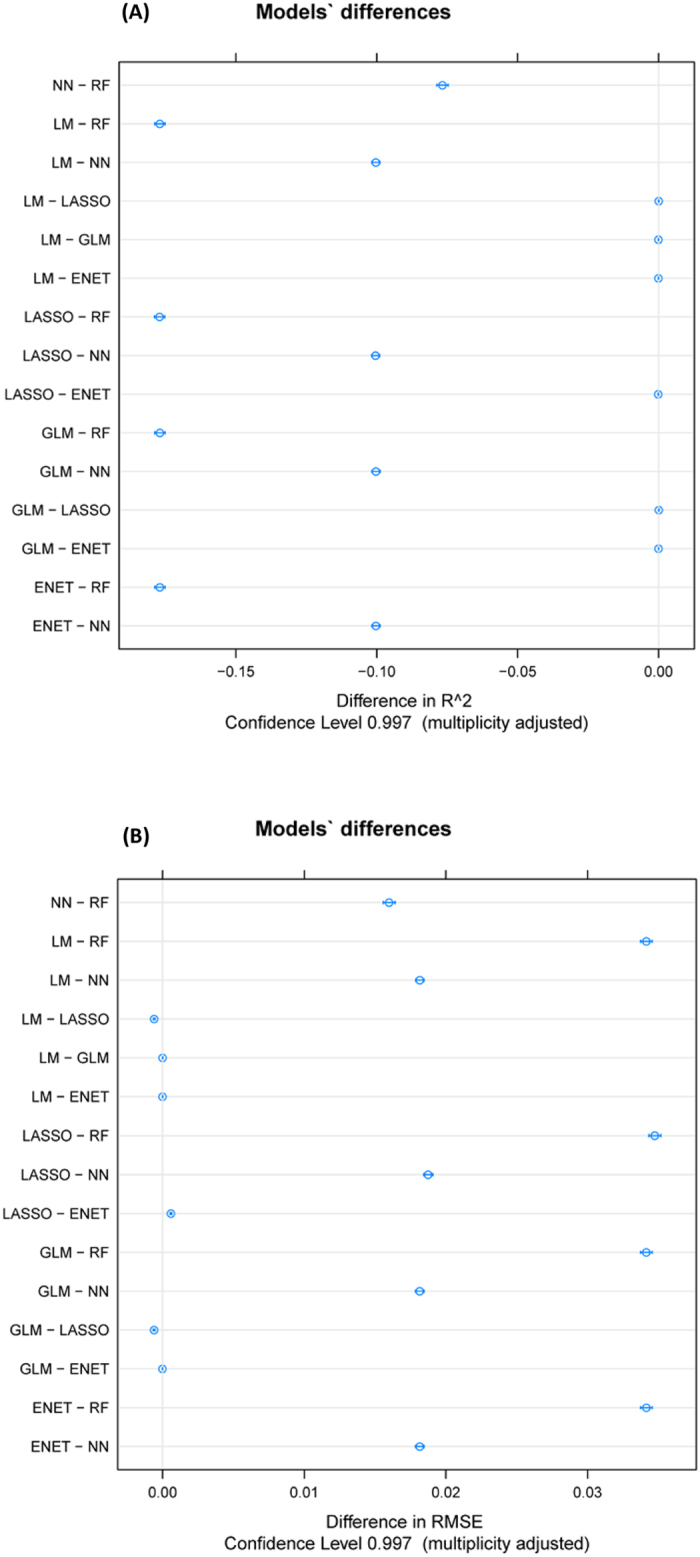
RRegrs pairwise model comparisons of R^2^_test_ and RMSE_test_. The average performance value (dot) with two-sided confidence limits as computed by Student’s t-test with Bonferroni multiplicity correction.

In the second step, the filtered dataset was processed with seven RRegrs methods: LM, GLM, PLS, Lasso, ENET, NN, and RF (Table 7). In addition, Fig. 4 presents the pairwise model comparisons of R^2^_test_ (A) and RMSE_test_ (B). The average performance value (dot) with two-sided confidence limits was computed using Student’s t-test with Bonferroni multiplicity correction. Even if the LM, GLM, PLS and ENET improve the model performance with over 0.04 of R^2^_test_ compared to the non-filtered dataset; the results are still moderate with the R^2^_test_ very close to 0.60. Lasso failed to select the system perturbation, in agreement with the previous results.

**Figure 5:**
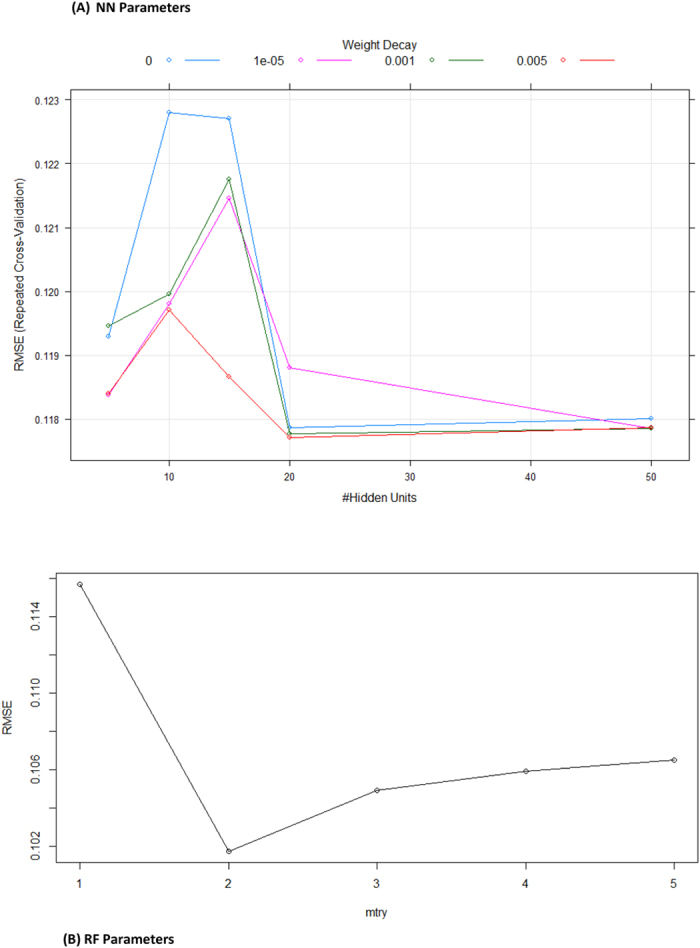
Variation of RMSE of two models with (**A**) the number of hidden layer neurons & weight decay of NN and (**B**) the number of features to feed the trees in RF.

NN model makes a clear difference with R^2^_test_ of 0.702 and RMSE_test_ = 0.117. NN has the topology of 5-20-1 (5 inputs, 20 neurons in one hidden layer, weight decay = 0.005). Figure 5A presents the NN parameter study using 5, 10, 15, 20 and 50 hidden layer neurons and weight decays of 0.0, 0.0001, 0.001 and 0.005. This performance is similar to the one obtained with MLP from STATISTICA (R^2^_test_ = 0.704).

The best model to predict growth yield (Y_pred_) was provided by RF method based on all features and five trees, with R^2^_test_ of 0.781 and RMSE_test_ of 0.101. By decreasing the number of trees to one, R^2^_test_ is lower (0.749) and RMSE_test_ is higher (0.109). Thus, the increase of the RF model complexity from one to five trees is needed to improve the regression performance. If the number of trees is increased to 10, R^2^_test_ becomes higher, with a value of 0.783 and RMSE_test_ lower (0.100). The difference between our best RF model with five trees and the one with ten trees is not in agreement with the increase of the model complexity. Thus, no further numbers of trees were tested with RF. The model can be downloaded from Figshare (SM04^56^).

Thus, the prediction regression model is constructed using mRNA expression in different parts of the GIT (spatial variation) and at different time-points (time variation). These are the spatiotemporal variations of the mRNA expression. The model input variables are MA values that are calculated as differences of the original values and the averages of the variables in eight different segments of the GIT, seven different sampling time points and two different feeding systems. The best final model could be used to predict the growth yield using new Ghrelin and Growth mRNA expressions measured under the model experimental conditions: one of the eight segments of the GIT, one of the seven sampling time points and one of the two feeding systems. The model input features as MA values will be calculated using the model averages of *Ghrelin* and *Growth* mRNA expressions from the training dataset. Introducing these MA values in the RF model, the growth yield can be predicted.

In conclusion, the current study investigated the tissue distribution and sequential dynamic developmental changes of *Ghrelin* and *GHR* mRNA expression. The factors of spatiotemporal development of GIT were taken into account, along with Supplemental feeding vs Grazing feeding systems, and new Machine Learning models were developed in order to predict the growth yield depending on the mRNA expression of *Ghrelin* and *GHR* after perturbations/variations of different conditions. Using linear and non-linear Machine Learning methods, it was found that the Random Forest method provided the best regression prediction model with R^2^_test_ > 0.78. In addition, this model also revealed that the mRNA expression of *GHR* could positively reflect the rate of growth yield, and it is crucial during the processes of growth and development in ruminants.

## Materials and Methods

### Experimental Animal and Management

All procedures for animal experimentation were carried out in accordance with the guidelines approved by the Animal Care Committee (Approval Number: 20130108), Institute of Subtropical Agriculture, the Chinese Academy of Sciences, Changsha, China. The principles of laboratory animal care were met and slaughter procedures were conducted according to the guidelines of Chinese national standards of cattle and goat slaughtering by reducing the animal suffering as much as possible. All experimental protocols were also approved by Institute of Subtropical Agriculture, the Chinese Academy of Sciences, Changsha, China.

A number of 44 newly born male and female kids (weighing an average of 1.35 ± 0.12 kg) were used as experimental animals to investigate the effect of age and feeding system (Supplemental vs Grazing, S vs G) on the expression of *Ghrelin* and *GHR* in the GIT of kid goats. After birth, the kids were separated from the dams and trained to suckle milk from nipple pails. Detailed feeding management, ingredients of concentrate starter and forage (mainly Miscanthus) have been described in our previous parallel study^27^. All goats had free access to water.

### Sample Collection

Mucosa samples of different GIT longitudinal segments (i.e., rumen, abomasum, duodenum, jejunum, ileum, colon, cecum, and rectum) were collected immediately after slaughter. The collected samples were wrapped with sterilized tinfoil and snap-frozen in liquid nitrogen and stored at −80 °C until RNA isolation.

### RNA Isolation and cDNA Preparation

Total RNA was extracted from collected mucosa samples using TRIZOL (Invitrogen, California, USA) according to the manufacturer’s instructions. After genomic DNA was eliminated by digestion with DNase I (Thermo Scientific, Waltham, USA), the RNA quality and quantity were determined. Afterwards, 1 μg of the extracted RNA was reverse-transcribed to synthesize tissue specific cDNA using PrimeScript™ RT reagent Kit (Takara, Dalian, China) immediately. Briefly, a 20 μL reverse transcription mixture that contained 1 μg of total RNA, 2 μL 5 × gDNA Eraser Buffer, 4 μL 5 × PrimeScript Buffer, 1 μL PrimeScript RT Enzyme Mix, 1 μL RT Primer Mix and 10 μL RNase Free dH_2_O was prepared. This reaction mixture was incubated for 2 min at 42 °C, followed by a reverse transcription step for 15 min at 37 °C, and a final heating step at 85 °C for 5 s to stop the reaction. The prepared cDNA samples were stored at −20 °C until subsequent quantitative real-time PCR analysis.

### Primer Design

Primers for quantitative real-time PCR analysis were designed according to *Ghrelin* (Accession No.: AB089200) and *GHR* (Accession No.: NM_001285648) gene sequences of *Capra hircus* reported online. β-Actin (Accession No.: NM_001009784.1) was used as a housekeeping gene in quantitative real-time PCR analysis. All primers were synthesized by Sangon Biotech (Sangon Biotech, Shanghai, China), and the primer sequences are shown in Table 8.

### Quantitative Real-Time PCR Analysis

The quantitative real-time PCR was performed on an ABI-7900HT qPCR system (Applied Biosystems, Foster City, CA, USA) using FG POWER SYBR GEREEN PCR MASTER MIX (Applied Biosystems, Foster City, CA, USA). The quantification of the PCR products of *Ghrelin* and *GHR* genes was evaluated in comparison with the PCR products of β-actin. The relative changes in mRNA expression levels determined from qPCR were calculated according to the 2^−△△CT^ method^57^, where −ΔΔCT = −(ΔCT _other tissue samples_ − ΔCT _duodenum sample at d0_) and ΔCT = CT _samples_ − CT _β-actin_.

### Statistical Analysis

The effect of the feeding system (S vs G) on the expression of *GHR* and *Ghrelin* was examined from time points d 28 to 70. The data were analyzed as a completely randomized design with the MIXED procedures of SAS (SAS Inst. Inc., Cary, NC) with a model that included the fixed effect of feeding system, age, and the feeding system × age interaction, with an individual animal as the experiment unit, as described in our previous study^27^. Orthogonal contrasts were used to test the linear and quadratic effects of age. The effects of age was tested with animal nested within age as the random effect and individual animal as the experimental unit. Statistical significance was defined as *P* < 0.05. The advantage of MIXED from general linear model (GLM) is it can handle correlated data and unequal variances, and it encompasses all models in the variance components procedure. In the linear mixed-effects model, the responses from a subject are the sum of fixed and random effects. The fixed effect affects the population mean and the random effect is associated with a sampling procedure. Another difference between MIXED and GLM is that MIXED is based on maximum likelihood (ML) and restricted maximum likelihood (REML) methods, while GLM uses the analysis of variance (ANOVA) methods. ANOVA can deal with balanced designs, whereas ML and REML are efficient with balanced and unbalanced designs (modeling real data). The full dataset with the experimental results is available as SM01^41^.

### Machine Learning Models

In the first step, the raw dataset was used to create the corresponding normalized dataset and the training and test sub-sets (using an R script): 75% training set (train) and 25% test set (test) (SM02^47^). The datasets and the R script are available in Figshare (SM04)^56^.

Two pieces of software were used to build regression models: STATISTICA and RRegrs. With STATISTICA multilinear and neural network regressions were used. The resulting models were characterized by the R_test_ values (regression coefficient for test subset). In addition, the corresponding R^2^_test_ values were added to the standard STATISTICA outputs using R_test_.

RRegrs is an R integrated framework used to create ten linear and non-linear regression models^45^,^46^. Due to the computational limitations generated by the big datasets, only seven RRegrs methods were used: Multiple Linear regression (LM), Generalized Linear Model with Stepwise Feature Selection (GLM)^50^, Partial Least Squares Regression (PLS)^51^, Lasso regression (Lasso)^52^, Elastic Net regression (ENET)^53^, Neural Networks regression (NN)^54^, and Random Forest (RF)^55^ (SM03^48^). In general, default values of specific parameters were used for the regression methods. For some procedures, particular variations of the method parameters were studied in order to provide the best possible regression model. The standard RRegrs call is not prepared for big datasets. Thus, individual RRergrs methods were used with only one training/test split, without several RRegrs features (Y randomization, RRegrs plotting, data scaling, near-to-zero variance filtering, feature correlation removal), and using specific method calls (RRegrs::Method, where Method is the name of the regression function in RRegrs). The graphics were constructed externally by saving the generated model objects. The criteria to find the best model are the same as for RRegrs: maximum R^2^_test_ and minimum RMSE_test_. The dataset splitting and the RRegrs results can be reproduced by using the same parameters and values of the seeds in the scripts. The best model available as Figshare item can be downloaded and studied with R for other statistics (SM04)^56^.

In order to compare them with the STATISTICA results, additional R.ts values were calculated as *sqrt*(R^2^_test_). Thus, in the results from STATISTICA and RRegrs, both R and R^2^ values have been reported. The importance of the features for the RRegrs models was calculated with *caret* functions *varImpPlot*(fited.model) and varImp(fited.model), where “fited.model” is the fitted model for the regression methods. The residual plot to remove the outliers and the best model (RF) are available in Figshare (SM04)^56^.

## Additional Information

**How to cite this article**: Ran, T. *et al*. Gastrointestinal Spatiotemporal mRNA Expression of *Ghrelin* vs *Growth Hormone Receptor* and New Growth Yield Machine Learning Model Based on Perturbation Theory. *Sci. Rep.* **6**, 30174; doi: 10.1038/srep30174 (2016).

## Figures and Tables

**Table 1:** The effect of Supplemental vs Grazing feeding system on the mRNA expression of *Ghrelin* and tissue distribution and dynamic developmental changes of *Ghrelin* mRNA expression during different stages of development.

Item	System	Age (d)							Development stage (age), day			*SEM*^*1*^	*SEM*^*2*^	*SEM*^*3*^	*P* value^1^			*P value for Age*^*2*^		*P* value^3^
		0	7	14	28	42	56	70	pre-rumination(d 0–14)	Transition(d 28–42)	rumination(d 56–70)				System	Age	System×Age	L	Q	
Rumen	S	0.004	0.003	0.002	0.002	0.004	0.000	0.002	0.003^a^	0.003^a^	0.002^a^	0.001	0.001	0.0004	0.1402	0.1178	0.8144	0.6250	0.5293	0.690
	G				0.002	0.004	0.003	0.004												
Abomasum	S	1.80	31.15	49.92	61.64	252.26	438.34	567.67	27.62^Ab^	176.76^Ab^	549.96^Bb^	48.901	46.67	73.207	0.0107	<0.0001	0.5194	<.0001	0.0002	<0.001
	G				85.12	308.01	564.58	629.26												
Duodenum	S	1.00	1.11	1.33	2.22	0.74	1.46	0.99	1.15^a^	2.04^a^	1.33^a^	0.286	0.293	0.202	0.0002	0.0006	<0.0001	0.7773	0.0094	0.168
	G				2.20	2.98	1.30	1.57					0.258					0.0045	<0.0001	
Jejunum	S	0.13	0.21	0.36	0.31	0.16	0.17	0.18	0.23^a^	0.31^a^	0.23^a^	0.064	0.073	0.029	0.0006	0.0141	0.5978	0.6901	0.0009	0.493
	G				0.43	0.34	0.33	0.24												
Ileum	S	0.03	0.02	0.001	0.00	0.01	0.01	0.02	0.02^a^	0.01^a^	0.01^a^	0.003	0.005	0.003	0.3724	0.0004	0.0048	0.1017	<0.0001	0.386
	G				0.01	0.01	0.01	0.01					0.005					0.0012	0.0005	
Cecum	S	0.01	0.03	0.05	0.02	0.01	0.01	0.01	0.03^a^	0.02^a^	0.02^a^	0.011	0.014	0.004	0.2033	0.2826	0.3089	0.0592	0.1091	0.341
	G				0.03	0.01	0.03	0.01												
Colon	S	0.02	0.04	0.03	0.05	0.05	0.05	0.05	0.03^a^	0.05^a^	0.05^a^	0.012	0.012	0.003	0.5923	0.4484	0.4493	0.0066	0.5921	0.121
	G				0.05	0.04	0.04	0.06												
Rectum	S	0.02	0.01	0.02	0.03	0.06	0.04	0.02	0.02^a^	0.05^a^	0.09^a^	0.022	0.011	0.014	0.0002	0.0161	0.0017	0.0734	0.0048	0.138
	G				0.05	0.04	0.15	0.14					0.025					<0.0001	0.0953	
SEM^4^									2.389	12.439	32.948									
*P* value^4^									0.012	<0.001	<0.001									

SEM^1^ represents standard error of mean for System × Age (from 28 to 70 d of age) on *Ghrelin* expression; *P* value^1^ represents *P* value for both treatment groups from 28 to 70 d of age on *Ghrelin* expression; SEM^2^ and *P* value for age^2^ represent SEM and *P* value for age from 0 to 70 d; SEM^3^ and *P* value^3^ represent SEM and *P* value for relative *Ghrelin* expression values at different development stages; SEM ^4^ and P value^4^ represent SEM and P value for different tissues at each developing stage. ^A,B^Means in the same row not bearing a common superscript letter differ (P < 0.05); ^a–c^Means in the same column not bearing a common superscript letter differ (P < 0.05); S, supplemental feeding; G, grazing; L = Linear effect of age, Q = Quadratic effect of age.

**Table 2:** The effect of Supplemental vs Grazing feeding system on the mRNA expression of growth hormone receptor (*GHR*) and tissue distribution and dynamic developmental changes of *GHR* mRNA expression during different stages of development

Item	System	Age (d)							Development stage (age), day			*SEM*^*1*^	*SEM*^*2*^	*SEM*^*3*^	*P* value^1^			*P value for Age*^*2*^		*P* value^3^
		0	7	14	28	42	56	70	pre-rumination(d 0–14)	Transition(d 28–42)	rumination(d 56–70)				System	Age	System×Age	L	Q	
Rumen	S	0.26	0.31	0.46	0.37	0.15	0.03	0.11	0.34^Ba^	0.15^Aa^	0.06^Aa^	0.058	0.057	0.048	<0.0001	0.0014	0.0008	<0.0001	0.0886	0.030
	G				0.03	0.03	0.03	0.05					0.017					<0.0001	<0.0001	
Abomasum	S	0.85	1.08	1.16	1.12	0.64	0.47	0.46	1.03^Bb^	0.67^ABb^	0.38^Ac^	0.157	0.184	0.101	0.0002	0.0004	0.1193	<0.0001	0.2788	0.014
	G				0.52	0.41	0.33	0.24												
Duodenum	S	1.00	0.75	1.02	0.97	0.47	0.49	0.45	0.92^Bb^	0.47^ABab^	0.31^Abc^	0.106	0.152	0.106	<0.0001	0.0002	0.0366	0.0004	0.7664	0.039
	G				0.28	0.15	0.17	0.11				0.131	<0.0001					0.0093		
Jejunum	S	1.21	0.73	0.77	0.22	0.36	0.31	0.27	0.90^Bb^	0.21^Aa^	0.23^Aab^	0.053	0.086	0.106	<0.0001	0.0703	0.2069	<0.0001	<0.0001	0.001
	G				0.11	0.13	0.16	0.16												
Ileum	S	0.89	0.55	0.25	0.15	0.15	0.27	0.12	0.56^Bab^	0.13^Aa^	0.15^Aab^	0.047	0.055	0.075	0.0010	0.0285	0.1185	<0.0001	<0.0001	0.017
	G				0.10	0.10	0.11	0.09												
Cecum	S	0.43	0.17	0.33	0.19	0.11	0.10	0.12	0.31^Ba^	0.11^Aa^	0.09^Aa^	0.028	0.046	0.037	<0.0001	0.0214	0.2815	<0.0001	0.0005	0.011
	G				0.08	0.05	0.06	0.07												
Colon	S	0.49	0.33	0.44	0.80	0.52	0.41	0.33	0.42^a^	0.52^ab^	0.30^bc^	0.121	0.139	0.049	0.0005	0.0016	0.1871	0.1233	0.0423	0.169
	G				0.38	0.36	0.29	0.16												
Rectum	S	0.39	0.45	0.31	0.40	0.57	0.34	0.33	0.38^a^	0.37^ab^	0.29^bc^	0.082	0.103	0.032	0.0007	0.3017	0.0959	0.1571	0.8832	0.483
	G				0.29	0.20	0.22	0.26												
SEM^4^									0.065	0.048	0.025									
*P* value^4^									<0.001	0.004	0.001									

SEM ^1^ represents SEM for System × Age (from 28 to 70 d of age) on *GHR* expression; *P* value^1^ represents *P* value for both treatment groups from 28 to 70 d of age on *GHR* expression; SEM^2^ and *P* value for age^2^ represent SEM and *P* value for age from 0 to 70 d; SEM^3^ and *P* value^3^ represent SEM and *P* value for relative *GHR* expression values at different development stages; SEM^4^ and *P* value^4^ represent SEM and *P* value for different tissues at each developing stage. ^A,B^Means in the same row not bearing a common superscript letter differ (P < 0.05); ^a–c^Means in the same column not bearing a common superscript letter differ (P < 0.05); S, supplemental feeding; G, grazing; L = Linear effect of age, Q = Quadratic effect of age.

**Table 3:**
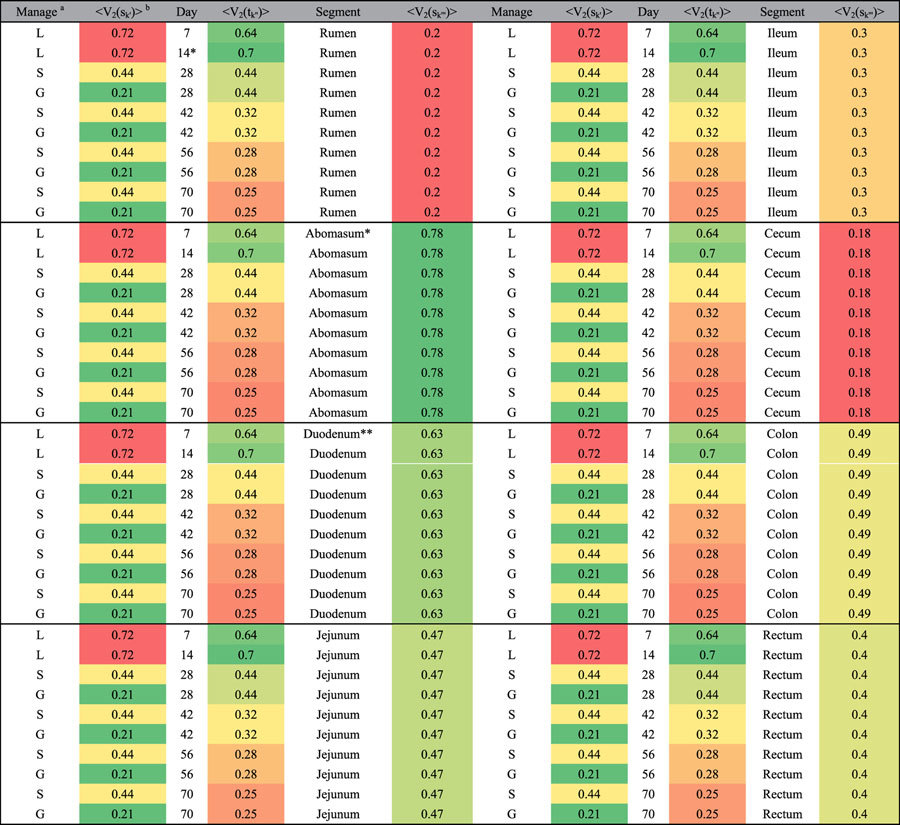
Expected values <V_2_(ζ_k_)> of *GHR* mRNA expressions corresponding to growth yields.

^a^Lactation represents the suckling periods of the goats (0–20 d), Housing refers to the goat with housing feed management, Grazing refers to the goats with grazing feed management. ^b^The green color means the strong/positive effect on growth yields, whereas, red color represents the poor/negative effect on growth yields. ^*^The mark *means the mRNA expression of *GHR* under this condition corresponds to the higher growth yields. L, lactation; S, supplemental feeding; G, grazing.

**Table 4:** Best regression models using neural network regression from STATISTICA with the normalized dataset.

Model	Error Mean	Error S.D.	Abs E. Mean	S.D. Ratio	R_test_	R^2^_test_*
LNN 2:2–1:1	0.0005	0.0992	0.0617	0.6827	0.731	0.534
LNN 5:5–1:1	0.0005	0.0992	0.0617	0.6828	0.731	0.534
MLP 2:2-7-1:1	−0.0290	0.0993	0.0634	0.6837	0.730	0.534
MLP 5:5-5-1:1	0.0084	0.0990	0.0636	0.6810	0.733	0.537
MLP 2:2-10-9-1:1	−0.9935	0.0998	0.9935	0.6868	0.727	0.529
**MLP 5:5-15-12-1:1**	−0.3918	0.0997	0.3918	0.6861	**0.734**	**0.539**

Note: LNN = Linear Neural Network; MLP = Multilayer perceptron; R_test_ = regression coefficient for test subset from STATISTICA; R^2^_test_ = coefficient of determination, calculated using R_test_.

**Table 5:** Best regression models using RRegrs package with normalized dataset.

RRegrs Method	No. of Features	Model Features	RMSE_train_	R^2^_train_	RMSE_test_	R^2^_test_	R_test_
LM	5	Pool	0.0995	0.537	0.0992	0.534	0.731
GLM	5	Pool	0.0995	0.537	0.0992	0.534	0.731
PLS	5	Pool	0.0996	0.536	0.0993	0.533	0.730
Lasso	1	Y_exp_	0.0998	0.537	0.0994	0.534	0.731
ENET	2	Y_exp_ + V_1_	0.0995	0.537	0.0992	0.534	0.731
NN	5	Pool	0.099	0.541	0.0986	0.540	0.735
**RF**	5	Pool	0.0881	0.638	**0.0886**	**0.629**	0.793

Note: LM = Multiple Linear regression; GLM = Linear Model with Stepwise Feature Selection; PLS = Partial Least Squares Regression; Lasso = Lasso regression; ENET = Elastic Net regression; NN = Neural Networks regression; RF = Random Forest; Pool = all five features, RMSE = root-mean-square error; R^2^ = coefficient of determination; R = regression coefficient, calculated as *sqrt*(R^2^); train = training subset; test = test subset.

**Table 6:** The best regression models using STATISTICA with filtered normalized dataset.

Model	Error Mean	Error S.D.	Abs E. Mean	S.D. Ratio	R_test_	R^2^_test_*
LNN 2:2-1:1	−0.001159	0.136078	0.105028	0.632973	0.774	0.599
LNN 5:5-1:1	−0.001160	0.136079	0.105029	0.632978	0.774	0.599
MLP 2:2-11-1:1	0.026576	0.117859	0.096420	0.548228	0.839	0.704
**MLP 5:5-6-1:1**	−0.307728	0.117102	0.307728	0.544707	0.839	**0.703**
MLP 2:2-5-6-1:1	−0.035073	0.117304	0.087202	0.545646	0.838	0.702
MLP 3:3-10-6-1:1	−0.004027	0.124694	0.094062	0.580020	0.815	0.664

Note: LNN = Linear Neural Network; MLP = Multilayer perceptron; R_test_ = regression coefficient for test subset from STATISTICA; R^2^_test_ = coefficient of determination, calculated using R_test_.

**Table 7:** RRegrs models using filtered normalized dataset.

RRegrs Method	No. of Features	Model Features	RMSE_train_	R^2^_train_	RMSE_test_	R^2^_test_	R_test_
LM	5	Pool	0.136	0.598	0.136	0.599	0.774
GLM	5	Pool	0.136	0.598	0.136	0.599	0.774
PLS	5	Pool	0.136	0.596	0.136	0.598	0.773
Lasso	1	Y_exp_	0.136	0.598	0.137	0.599	0.774
ENET	2	Y_exp_ + V_1_	0.136	0.598	0.136	0.599	0.774
NN	5	Pool	0.118	0.698	0.117	0.702	0.838
**RF**	5	Pool	0.102	0.775	**0.101**	**0.781**	0.884

Note: LM = Multiple Linear regression; GLM = Linear Model with Stepwise Feature Selection; PLS = Partial Least Squares Regression; Lasso = Lasso regression; ENET = Elastic Net regression; NN = Neural Networks regression; RF = Random Forest; Pool = all five features, RMSE = root-mean-square error; R^2^ = coefficient of determination; R = regression coefficient, calculated as *sqrt*(R^2^); train = training subset; test = test subset.

**Table 8:** Details of primers used in the current study.

Gene	NCBI (Access No.)	Primer Sequence (5′-3′)	Product Size (bp)	Annealing Temperature (^o^C)
*GHR*	NM_001285648	F: GGAACCACCACCCAATACAG	167	60
		R: TCACACGCACTTCATACTCCTT		
*Ghrelin*	AB089200	F: GCGGGCTCCAGCTTTCTGAG	110	60
		R: TCCGGGTCAAACTGGCCTTC		
*β-Actin*	NM_001009784.1	F: CTTCCAGCCTTCCTTCCTG	111	60
		R: ACCGTGTTGGCGTAGAGGT		
